# Effects of the long-term influence of bisphenol A and bisphenol S on the population of nitrergic neurons in the enteric nervous system of the mouse stomach

**DOI:** 10.1038/s41598-023-27511-9

**Published:** 2023-01-06

**Authors:** Krystyna Makowska, Jarosław Całka, Sławomir Gonkowski

**Affiliations:** 1 Department of Clinical Diagnostics, Faculty of Veterinary Medicine, University of Warmia and Mazury in Olsztyn, Oczapowskiego 14, 10-957 Olsztyn, Poland; 2 Department of Clinical Physiology, Faculty of Veterinary Medicine, University of Warmia and Mazury in Olsztyn, Oczapowskiego 13, 10-957 Olsztyn, Poland

**Keywords:** Cell biology, Environmental sciences, Natural hazards

## Abstract

Bisphenol A (BPA) is an endocrine disruptor commonly used in the production of plastics. Due to its relatively well-known harmful effects on living organisms, BPA is often replaced by its various analogues. One of them is bisphenol S (BPS), widely used in the plastics industry. Until recently, BPS was considered completely safe, but currently, it is known that it is not safe for various internal organs. However, knowledge about the influence of BPS on the nervous system is scarce. Therefore, the aim of this study was to investigate the influence of two doses of BPA and BPS on the enteric nitrergic neurons in the CD1 strain mouse stomach using the double-immunofluorescence technique. The study found that both substances studied increased the number of nitrergic neurons, although changes under the impact of BPS were less visible than those induced by BPA. Therefore, the obtained results, for the first time, clearly indicate that BPS is not safe for the innervation of the gastrointestinal tract.

## Introduction

Bisphenols are a group of substances widely used in the chemical industry to produce polycarbonate plastics and epoxy resins, and therefore they are present in various everyday objects, including bottles, thermal paper, and food can linings^1,2^. Until recently, bisphenol A (BPA) was the most commonly utilised substance in industry from the group of bisphenols^3,4^. However, it has been shown that BPA, mainly due to its estrogenic properties, has a strong negative influence on living organisms^3–5^. Therefore, numerous countries have introduced restrictions on the use of BPA, and this substance has been replaced by its analogues, especially in products which have contact with food and/or items intended for children^6–8^. One BPA analogue that is gaining increasing popularity is bisphenol S (BPS). Until recently, BPS was considered completely safe for living organisms. However, recent studies have shown that since this substance, similarly to BPA, stimulates estrogenic receptors, it may negatively impact a wide range of physiological processes^9–12^ and even promote the progression of cancers^13,14^. Notwithstanding the constantly evolving knowledge on the impact of BPS on human and animal organisms, there is a lack of information regarding many aspects of this impact. One of the gaps in information concerns the influence of BPS on the enteric nervous system (ENS), although bisphenols most often enter living organisms via the gastrointestinal (GI) tract, and ENS is one of the first barriers against toxic substances^15^.

The structure of the ENS differs depending on the animal species as well as the part of the GI tract studied^16–20^. In the mouse stomach, it is formed with two kinds of intramural ganglia: (1) myenteric ganglia (MG) situated between the longitudinal and circular muscle fibres, which are interconnected with a dense neuronal fibre network to form myenteric plexus and (2) submucous ganglia located in the submucosal layer, near the gastric mucosa^19,20^.

The ENS is characterised by significant variability in terms of the synthesis of neuromediators and/or neuromodulators. Enteric neurons may synthetize several dozen active substances, and the main reaction of the ENS to pathological stimuli (including the intoxication) involves changes in neuronal neurochemical characterisation, which means fluctuations in the expression (and release) of neuroactive factors^21,22^.

Among the neuronal factors produced by enteric neurons, the gaseous neurotransmitter nitric oxide (NO) is one of the most important factors in the regulation of gastrointestinal functions^23^. It is known that nitrergic neurons occur in the ENS of many species, including humans^20,24–27^. The number of these neurons differs depending on animal species, the segment of the GI tract and the type of enteric ganglia studied^20,25–29^. Moreover, in most cases, including the mouse stomach, the population of nitrergic enteric neurons is relatively numerous^20,28–31^.

Previous investigations have shown that NO is one of the most important inhibitory factors in the ENS and takes part in the regulation of several important functions of the GI tract^23^. In addition to the relaxation of the gastrointestinal smooth muscles and intramural and mesenteric vessels, which makes this neurotransmitter involved in the motility and intestinal blood flow regulations^32–35^, NO is also involved in the inhibition of the secretion processes in the stomach and intestines^26,28,36^.

Although there have been many studies on the role of NO in the ENS, some aspects of its activity are still not clear. Although it is suggested increasingly often in recent studies that this neurotransmitter is involved in the pathological and neuroprotective processes in the intestines, knowledge on this topic is still rather scarce and incomplete^27,28,37^.

Therefore, the aim of the present study was to compare, for the first time, the influence of long-term exposure to BPA and BPS on the population of nitrergic neurons in the stomach, which are responsible for storage, mixing and partial digestion of the food and whose proper functioning largely determines metabolism and absorption of toxins entering the living organism orally as well as the appropriate digestion of all food ingredients^15^.

Thus, the hypothesis proposed by the authors was that BPS (like BPA) also adversely affects the ENS, which is the first barrier that these toxins must overcome when entering the living organism via the gastrointestinal tract, and nNOS expression will significantly change as a result of the neuroplasticity of the nitrergic neurons.

## Results

**Figure 1:**
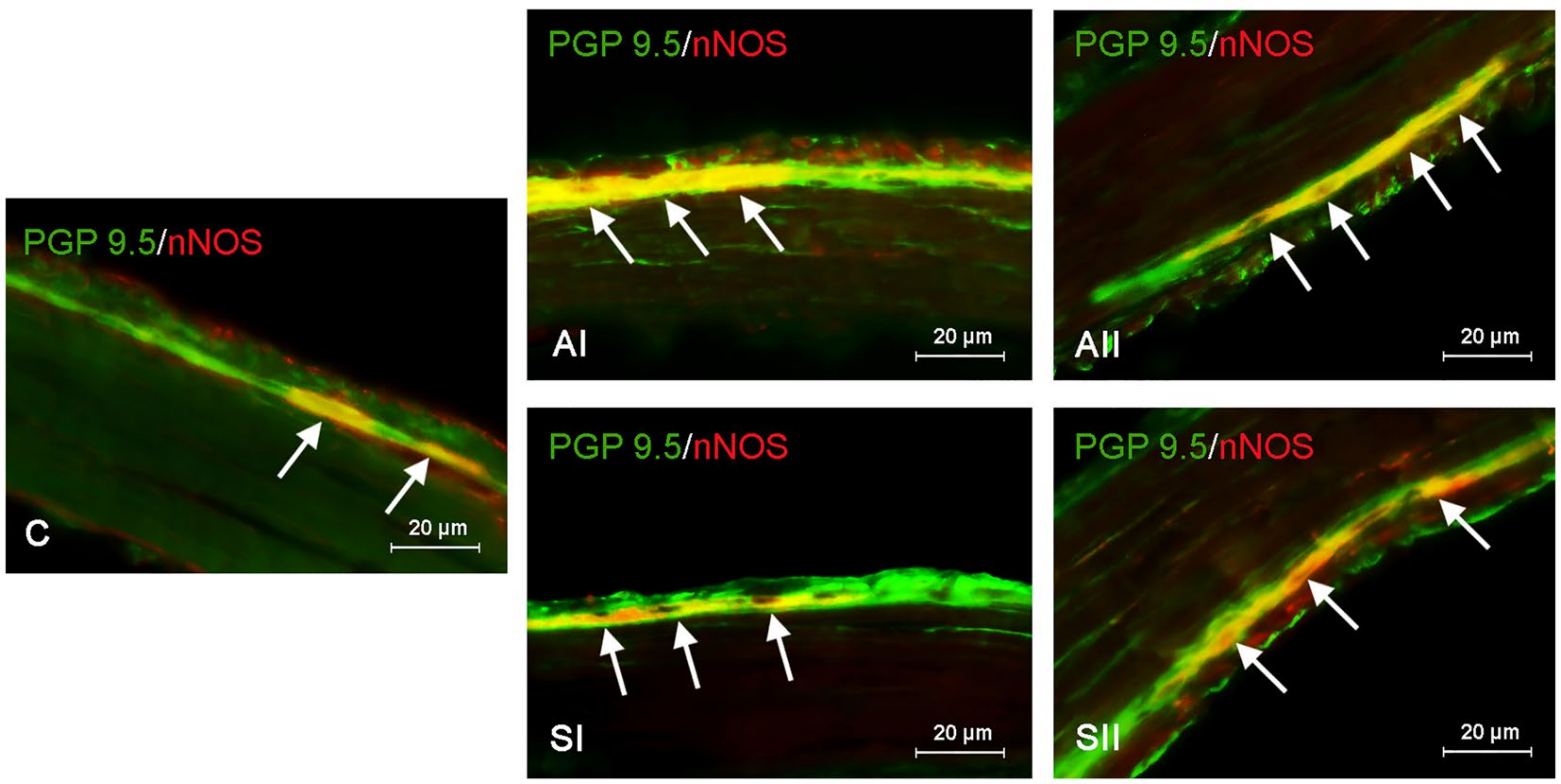
Distribution pattern of nerve cells immunoreactive to protein gene-product 9.5 (PGP 9.5)—used as a pan-neuronal marker and neuronal isoform of nitric oxide synthase (nNOS) in the myenteric plexus (MG) of the mouse stomach under physiological conditions (C) and after administration of bisphenol A in a lower (AI) and higher dose (AII) and bisphenol S in a lower (SI) and higher dose (SII). The pictures are the result of the overlap of both stainings. The arrows are pointing to neurons immunoreactive for both—PGP 9.5 and nNOS.

**Figure 2:**
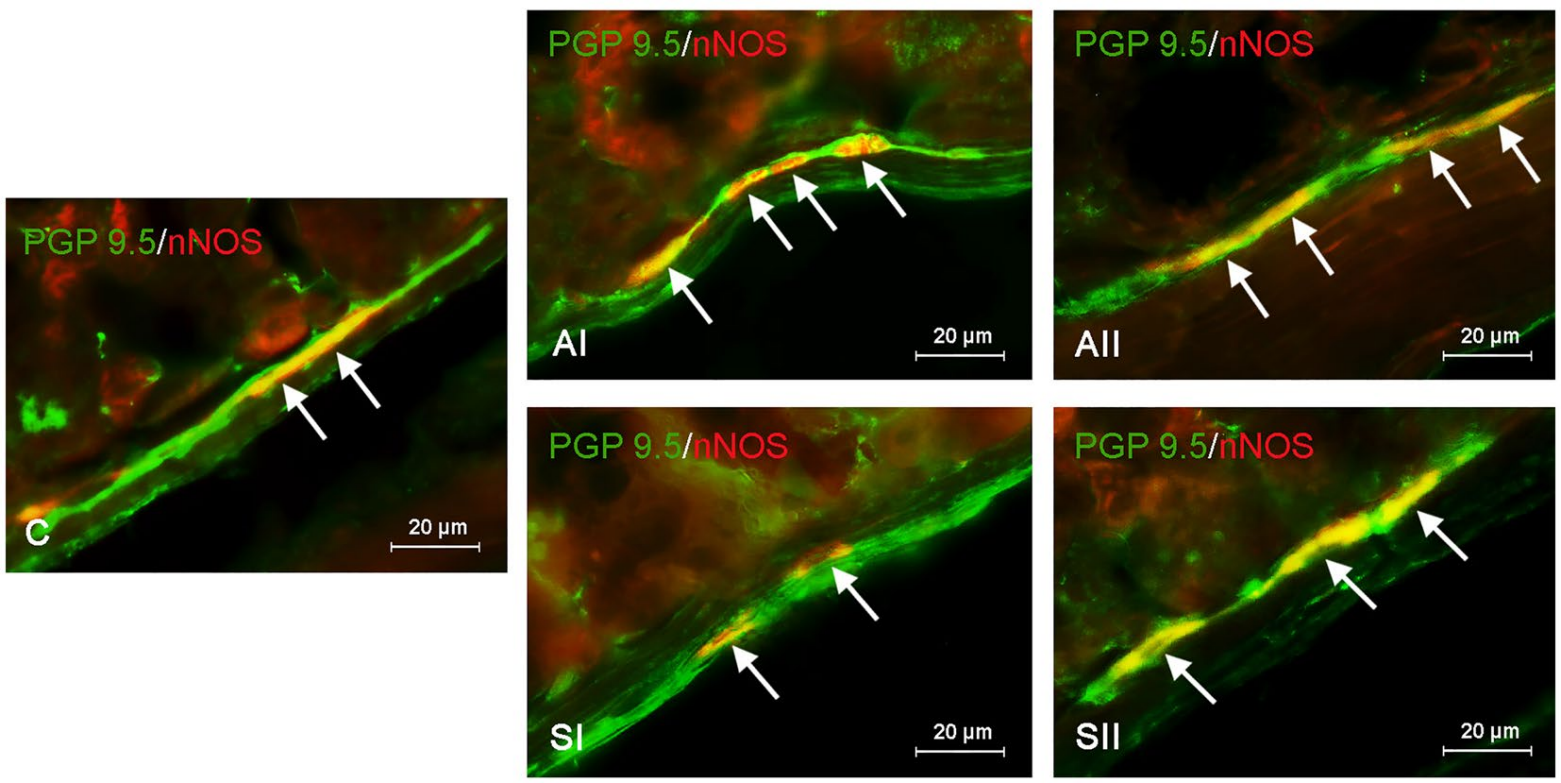
Distribution pattern of nerve cells immunoreactive to protein gene-product 9.5 (PGP 9.5)—used as a pan-neuronal marker and neuronal isoform of nitric oxide synthase (nNOS) in the submucous plexus (SG) of the mouse stomach under physiological conditions (C) and after administration of bisphenol A in a lower (AI) and higher dose (AII) and bisphenol S in a lower (SI) and higher dose (SII). The pictures are the result of the overlap of both stainings. The arrows are pointing to neurons immunoreactive for both—PGP 9.5 and nNOS.

In the present study, perikaryons positive to nNOS were observed in both the myenteric and submucous ganglia of the gastric ENS in all groups of animals (Figs. 1, 2).

In the control animals, the number of nNOS-positive neurons was up to 32.4 ± 0.566% and 30.58 ± 0.88% of all cells labelled with PGP 9.5 in the MG and SG, respectively (Table 1).

**Table 1:** The number of neurons positive for a neuronal isoform of nitric oxide synthase (nNOS) in the MG (myenteric plexus) and SG (submucous plexus) in the control group (C) and all of the experimental groups (AI, AII, SI, SII).

	C	AI	AII	SI	SII
MG	32.4 ± 0.566^a^	38.36 ± 0.813^a.b^	44.86 ± 0.735^a.c^	34.44 ± 0.6^a.b^	35.02 ± 0.885^a.c^
SG	30.58 ± 0.88^a^	38.49 ± 0.771^a.b^	38.14 ± 0.565^a.c^	31.36 ± 1.092^b^	34.02 ± 0.946^a.c^

Statistically significant (p ≤ 0.05) differences between group C and every experimental group were marked with ^a^, between the AI group and SI group were marked with ^b^, and between the AII group and SII group were marked with ^c^.

Both bisphenols used in the present study change the number of nNOS-positive neuronal cells in the MG and SG in the mouse stomach (Table 1). The degree of observed changes differs depending on the type of bisphenol studied, the type of enteric ganglion and the dose of substances.

In the case of BPA, the variations in the number of nNOS—immunoreactive neurons compared to the control group were most visible (Table 1). In the animals of the AI group, the percentage of perikaryons containing nNOS increased to 38.36 ± 0.813% (Fig. 1) and 38.49 ± 0.771% (Fig. 2) in the MG and SG, respectively. Interestingly, exposure to a higher dose of BPA (group AII) did not cause further growth of the number of nNOS-positive neurons in the SG, where the percentage of such neuronal cells was very similar to the results from the AI group and amounted to 38.14 ± 0.565% (Table 1). Contrary to SG, the percentage of nitrergic neurons in the MG under the impact of higher doses of BPA (group AII) was clearly higher than values noted after administration of lower dosages of BPA (group AI) and amounted to 44.86 ± 0.735% of all cells containing PGP 9.5 (Fig. 1).

Exposure to BPS also caused changes in the percentage of nNOS-positive enteric neuronal cells, but changes were less than those noted under the impact of BPA (Table 1). As in the case of BPA, BPS-induced changes also depended on both the dose of the substance and the type of enteric ganglion studied. The administration of a lower dose of BPS (SI group) led to a slight increase in the population of nNOS-positive neurons in the MG, where such neurons achieved all PGP 9.5—immunoreactive cells 34.44 ± 0.6% (Fig. 1). In turn, in the SG of animals treated with lower doses of BPS, the percentage of nitrergic neurons amounted to 31.36 ± 1092% of all neuronal cells labelled with PGP 9.5, and this value did not differ statistically significantly from that observed in control animals (Table 1). The fluctuations in a population of nNOS-immunoreactive perikaryons were more visible in the group of mice treated with a higher dose of BPS (SII group). In this group, the percentage of nitrergic neurons reached 35.02 ± 0.885% and 34.02 ± 0.946% in the MG and SG, respectively (Table 1).

## Discussion

During the present study, a relatively large population of nitrergic neurons in both types of enteric ganglia of the mouse stomach were noted under physiological conditions. These observations are in agreement with previous studies describing the nitrergic innervation in the ENS within the whole GI tract in many species, including humans^20,26,28,30,38–40^. Although the population of nNOS-immunoreactive neurons and nerve fibres in the gastric ENS in mammals has been previously well described^30,40–42^, it should be emphasised that the knowledge concerning the distribution of these nervous structures in the mouse stomach is rather scarce^19,42^.

The high amount of nitrergic neurons in the gastric ENS found in both the current study and previous studies is justified by the very important role of this neurotransmitter in the functioning of the GI tract. It is well known that NO is one of the most important inhibitory factors occurring in the ENS^23,43–49^. First of all, NO is known for its role in smooth muscle relaxation, local and systemic blood flow regulation and anti-inflammatory processes in the GI tract^32–35^. Moreover, the small size of this gaseous neurotransmitter allows it to pass through the cell membrane and manage the functions of the gastric and intestinal endocrine cells. In this way, NO can regulate the production of gastrin and affect stomach acid release^50^.

Nitric oxide is basically involved in the regulation of all gastric functions, such as storage, mixing and ingesting of the alimentary content. Moreover, previous studies have reported that NO plays some protective functions in the GI tract^32,35,51^. The gastroprotective role of this neurotransmitter has been noted in many pathological states, including inflammatory processes, chemically induced mucosal damage or intoxication^52–57^. However, it is known that NO not only can take part in anti-inflammatory processes but can also induce inflammation responses mostly because of its cytotoxic properties^55^.

Furthermore, in the mammal GI tract, according to the suggestions of previous findings, NO can have some neuroprotective role in the ENS^27,28,37,56^. Concerning these NO properties, it can be concluded that changes in the population of nNOS-positive neurons observed during the present study were connected with the neuroprotective functions of this neurotransmitter activated under the influence of both bisphenols studied.

Previous studies have relatively well documented the harmful effects of BPA, which causes changes in many systems and organs. Because of its similarities to oestrogen, BPA can combine with this hormone receptors, which are ubiquitous in the organisms of animals and humans^3,4^. Thus, BPA affects the reproductive, immune, gastrointestinal and nervous systems^3,4,28,57^ and, as a strong endocrine disruptor, it can lead to obesity, diabetes or hypertension^4,58^. It can also damage some parenchymatous organs like the liver or kidneys and even lead to their failure^4,59^. Several studies have also described the impact of BPA on the ENS and have shown many bisphenol-induced changes in the chemical coding of neurons and nerve fibres in the ENS of many parts of the GI tract^28,60–62^, which has also been confirmed in the present study, in which the influence of BPA on the enteric neurons in mouse has been described for the first time. Moreover, the impact of BPA on the enteric nitrergic neurons is generally similar in pigs^63^ and in mice (this study), which could suggest that BPA may affect the production of NO in the ENS regardless of mammal species, and similar effects may be expected in the case of the human GI tract.

Interestingly, the present research has also confirmed that not only BPA, but also BPS in a dose of 5 mg/kg b.w. may affect the number of nitrergic neurons in the ENS. The BPS-induced changes noted in this study were indeed less pronounced than those observed under the influence of BPA, but they were clearly visible, and their character (an increase in the number of neurons immunoreactive to nNOS) was similar under the impact of both bisphenols. It should be pointed out that, until recently, BPS (unlike bisphenol A) was considered safe for living organisms. For this reason, BPA has often been replaced by BPS in the production of plastics, especially in the case of items that come into contact with food or are intended for children.

Currently, an increasing number of studies have shown that BPS acts on living organisms similar to BPA, and its estrogenic effects may be even higher than the effects shown by BPA^64^. Moreover, some studies have shown that BPS may also show cytotoxic, genotoxic, neurotoxic, and immunotoxic responses^65,66^.

Despite these studies, BPS is even today often considered a good substitute for BPA in the production of plastics, and knowledge of the harmful effects of this substance is relatively scarce. One of the completely unknown aspects of the impact of BPS on a living organism is its effect on the ENS. The results obtained in the present study have shown for the first time that BPS may change the number of enteric nitrergic neurons. The present research found some statistically significant changes between groups of animals under the impact of BPS compared to those noted after BPA administration, which definitely shows that BPA has a stronger effect on the ENS in the mouse stomach. That is incompatible with some previous studies reporting that BPS shows a similar, or even stronger, effect than BPA on some parts of the living organism, such as the endocrine system^67^. However, both substances noticeably influenced the population of nitrergic neurons, and that observation is probably due to the structural similarities of these substances^64^, which show comparable influence not only in the term of estrogenic effects, cytotoxicity, genotoxicity and/or immunotoxicity^12–14,64,68,69^ but also regarding the impact on the ENS.

This may be all the more important as BPS is used instead of BPA, mainly in objects that come into contact with nourishment, and it first affects the digestive tract and ENS after entering the body with food.

It should be pointed out that the mechanisms of toxicity noted in the present study are not obvious. One of the possible reasons for the higher amount of enteric nitrergic neurons under the influence of bisphenols compared to the control animals may be connected with the above-mentioned neuroprotective properties of NO. On the other hand, the observed changes can also be associated with the relaxing effect of bisphenols on the gastrointestinal tissue^69^ because, as is well known, NO is involved in the relaxation mechanism of smooth muscles in the GI tract, including the stomach^34,35^. The next reason for the observed changes may be connected with the fact that BPA can modulate the number of calcium ions in neurons^57^. In turn, changes in calcium ions can lead to an increase in the synthesis of NO, which is connected with the cytotoxic properties of this gaseous factor in the cell as a free radical^55^. Thus, the observed increase in the number of nitrergic neurons during the present study may be a sign of some subclinical inflammatory changes in the stomach tissue.

The present study has also shown that even doses of BPA and BPS corresponding to the established NOAEL of BPA for mice may influence the number of enteric nitrergic neurons. It has been reported that bisphenols can lead to some long-term dysfunctions or even organ failure and cancer development^3,4^. Because of these properties during the present investigation, BPA and BPS have been given to the experimental animals for 3 months. Therefore, the intoxication effects observed in the study are probably caused by long-term exposure to these toxins. This particularly concerns small doses of bisphenols, which should not cause any changes in the organism after a short time of exposure.

To sum up, the obtained results have shown for the first time that not only BPA but also BPS may affect the number of nitrergic neurons in the ENS of the mouse stomach. Moreover, these changes have been noted under the impact of 5 mg/kg b.w. of bisphenols studied, which is the established NOAEL dose of BPA for mice. The present study strongly suggests that BPS, although until recently it was considered completely safe, is not really safe for living organisms. In turn, similarities in changes caused by BPS with those noted under the impact of BPA suggest comparable influences of both bisphenols on the enteric neurons. Although changes in the enteric nitrergic neurons noted in the present investigation may be connected with proinflammatory, cytotoxic and/or neurotoxic properties of bisphenols, the exact mechanisms of these changes are not quite clear, and clarification of all aspects connected with BPS-induced changes in the ENS requires further comprehensive research. 

## Materials and methods

### Animals and treatment

This study was performed on 35 adult mice (CD1 strain) of both genders and 30 g body weight. At the age of 3 months, animals were included in the study and, for the next 3 months of the investigation, were kept under standard laboratory conditions, including constant temperature (22 ± 20 °C) and humidity (55 ± 10%), 12:12 h light–dark cycle and access to water and food ad libitum. All procedures associated with the experiment have received the approval of the instructions of the Local Ethical Committee in Olsztyn (Decision No. 46/2019).

The animals were divided into five groups, with seven animals in each. The first group consisted of control mice (C group) without any experimental manipulations, and animals from the other four groups (groups AI, AII, SI and SII) were treated with BPA or BPS, given in the drinking water for 3 months in two doses: 5 mg/kg body weight (b.w.) or 50 mg/kg b.w. as presented in Table 2. The doses were adjusted to the amount of drinking water with the assumption that a mouse drinks about 2 ml/10 g b.w. per day^70^.

**Table 2:** Description of groups (dose and type of administrated substance) of animals used in the study. *BPA* bisphenol A, *BPS* bisphenol S, *C* control, *b.w.* body weight.

Group	C	AI	AII	SI	SII
Substance	X	BPA	BPA	BPS	BPS
Dose	X	5 mg/kg b.w.	50 mg/kg b.w.	5 mg/kg b.w.	50 mg/kg b.w.

Based on previous studies, the dose of 5 mg/kg b.w. is a no-observed-adverse-effect level (NOAEL) for BPA in mice, and the dose of 50 mg/kg b.w. is the lowest-observed-adverse-effect level (LOAEL) for BPA in mouse^71–73^. To compare the impact of both bisphenols, BPA and BPS were given in the same doses. The method of bisphenol administration was typical for this kind of experiment and was based on previous studies^68,74^. In order to give correct doses of bisphenols, according to the body weight, the animals were weighed once a week.

### Double-labelling immunofluorescence technique

After a period of 3 months, all animals were sacrificed (by decapitation). Immediately after the decapitation, whole stomachs were collected. The collected tissues were post-fixed by immersion with 4% buffered paraformaldehyde (pH 7.4) overnight, rinsed in phosphate buffer (0.1 M, pH 7.4, at 4 °C) for the next 3 days, put into an 18% phosphate-buffered sucrose solution and stored at 4 °C for at least 3 weeks. The tissues were then frozen at − 22 °C, cut into 10-μm-thick sections using a microtome (Microm, HM 525, Walldorf, Germany) and fixed on glass slides.

**Figure 3:**
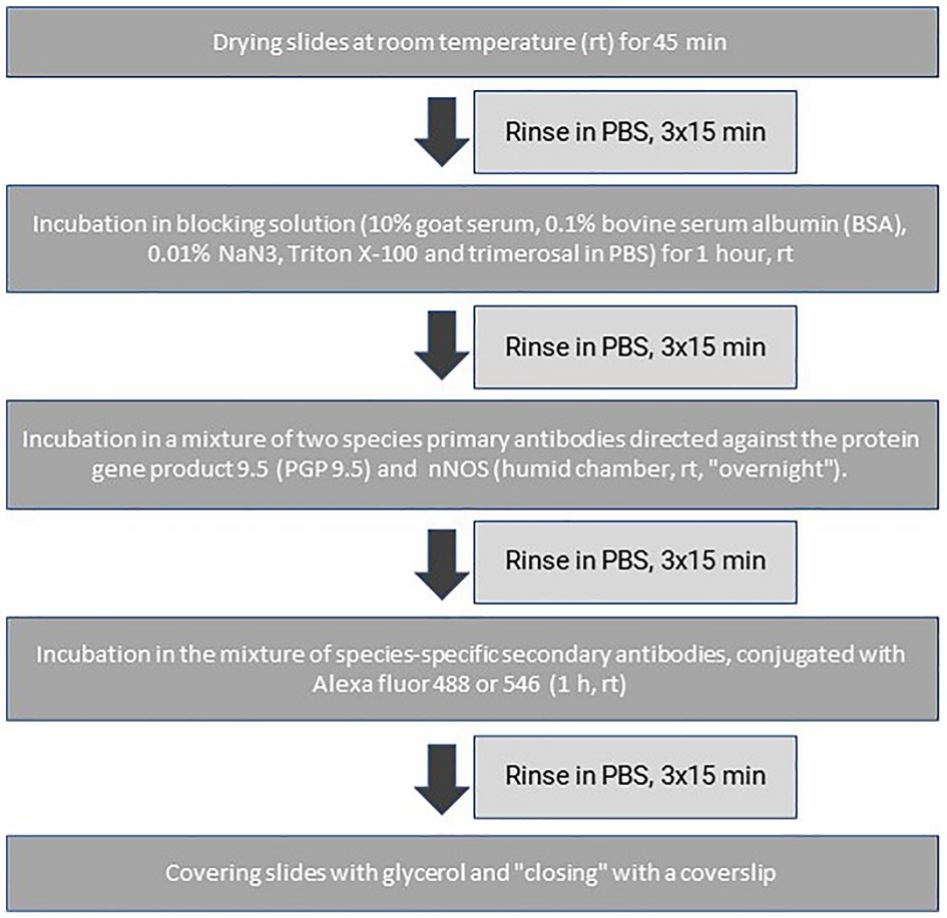
The scheme of labelling used during the present study. *PBS* phosphate buffered saline, *nNOS* neuronal isoform of nitric oxide synthase.

Afterwards, slides with fragments of the stomachs underwent the standard double labelling immunofluorescence technique as described previously by Gonkowski et al.^38^. For this purpose, two types of primary antibodies were used: mouse monoclonal antibody directed towards protein gene-product 9.5 (PGP 9.5, Biogenesis, UK, catalogue no 7863-2004, working dilution 1:1000, used here as a pan-neuronal marker) and rabbit polyclonal antibody towards neuronal isoform of nitric oxide synthase (nNOS, MercMillipore, DEU, catalogue no AB5380, working dilution 1:600, used here as a marker of nitrergic neurons). For the visualisation of “primary antibody–antigen” complexes, species-specific secondary antibodies were conjugated with appropriate fluorochromes (Alexa fluor 488 donkey anti-mouse IgG and Alexa fluor 546 donkey anti-rabbit IgG, Invitrogen, Carlsbad, CA, USA, working dilution 1:1000) were used. The scheme of labelling used during the present study is presented in Fig. 3.

During the present investigation, routine specificity tests of antibodies such as pre-absorption, omission and replacement of primary antibodies by non-immune sera were performed, and the results of these tests allowed to completely eliminate the nonspecific stainings.

Labelled fragments of the stomach were analysed with an Olympus BX51 microscope with epifluorescence and appropriate filter sets connected with an Olympus XM10 camera. To determine the percentage of nNOS-positive neurons, at least 500 cells immunoreactive to PGP-9.5 in the particular type of enteric ganglia from each animal were investigated for the occurrence of nNOS. The number of PGP-9.5-positive cells was regarded as 100%. For this purpose, only perikaryons with a clearly visible nucleus were accepted. The obtained results were pooled and presented as mean ± SEM. To avoid double counting the same neuronal cells, the fragments of stomachs included in the investigation were located at least 100 µm apart.

### Statistical analysis

For statistical analysis, the Anova test (Statistica 13, StatSoft, Inc., Cracow, Poland) was used, and the differences were considered statistically significant at p ≤ 0.05.

### Guidelines declaration statement

All experimental protocols were approved by the Local Ethical Committee for Animal Experiments in Olsztyn working at the University of Warmia and Mazury in Olsztyn, according to Act for the Protection of Animals for Scientific or Educational Purposes of 15 January 2015 (Official Gazette 2015, No. 266), applicable in the Republic of Poland (based on the consent No. 28/2013/N from May 22nd, 2013). During this experiment, all methods were carried out in accordance with relevant guidelines and European and Polish regulations. Moreover, the study was carried out in compliance with the ARRIVE guidelines.

## Data Availability

The datasets generated during and/or analyzed during the current study are available from the corresponding author on reasonable request.
